# Accuracy, rationality and specialization in a generalized model of collective navigation

**DOI:** 10.1098/rsif.2024.0207

**Published:** 2024-09-25

**Authors:** Richard P. Mann, Joseph D. Bailey, Edward A. Codling

**Affiliations:** ^1^ Department of Statistics, School of Mathematics, University of Leeds, Leeds, UK; ^2^ School of Mathematics, Statistics and Actuarial Science, University of Essex, Colchester, UK

**Keywords:** animal movement, collective navigation, leadership, many wrongs principle, rationality

## Abstract

Animal navigation is a key behavioural process, from localized foraging to global migration. Within groups, individuals may improve their navigational accuracy by following those with more experience or knowledge, by pooling information from many directional estimates (‘many wrongs’) or some combination of these strategies. Previous agent-based simulations have highlighted that homogeneous leaderless groups can improve their collective navigation accuracy when individuals preferentially copy the movement directions of their neighbours while giving a low weighting to their own navigational knowledge. Meanwhile, other studies have demonstrated how specialized leaders may emerge, and that a small number of such individuals can improve group-level navigation performance. However, in general, these earlier results either lack a full mathematical grounding or do not fully consider the effect of individual self-interest. Here we derive and analyse a mathematically tractable model of collective navigation. We demonstrate that collective navigation is compromised when individuals seek to optimize their own accuracy in both homogeneous groups and those with differing navigational abilities. We further demonstrate how heterogeneous navigational strategies (specialized leaders and followers) may evolve within the model. Our results thus unify different lines of research in collective navigation and highlight the importance of individual selection in determining group composition and performance.

## 1. Introduction

Navigation is important for diverse animal species across a range of different spatiotemporal scales, including key behaviours such as foraging and migration [1]. Understanding how animals are able to navigate accurately is hence a key open question in movement ecology [2,3]. Collective navigational benefits are observed across a variety of taxa [4], such as birds [5–8], fish [9,10], insects [11,12], mammals [13] and even humans [14]. Improved navigational accuracy may represent a powerful reason for these species to migrate collectively, alongside other factors such as predator avoidance or sharing information about resources [15]. However, the exact mechanisms for how information is shared across the group are not always well understood. Individuals may be naive, with little or no navigational information, but are able to follow other more experienced and knowledgeable individuals to reach a common target [16–18]. Alternatively, all group members may have limited navigational information and it is only through the pooling of many inaccurate individual directional estimates that the group as a whole is able to navigate, a mechanism known as the ‘many wrongs principle’ [19,20].

The simplest model for collective navigation under the ‘many wrongs’ paradigm assumes that all individuals receive noisy, but unbiased, estimates of the target, and the group has perfect integration of this information between individuals. Where individual estimates are also independent and identically distributed, the expected error of such a collective estimate straightforwardly follows from the central limit theorem [21]. However, these assumptions are overly simplistic for several reasons. First, the assumption that individuals contribute equally to collective navigation is violated in practice [8], and evolutionary modelling suggests that many group members may free-ride on the navigational efforts of a small proportion of the group [22]. Second, animals on the move are usually not able to easily indicate the precise information they hold and must instead arrive at a collective direction of motion by attending to the movements of others in the group, who in turn also attend to each other and to the focal individual [23]. As such, these movements cease to represent independent sources of information. The waggle dance of foraging bees is a notable exception, but one that takes place inside the hive and not during collective motion itself [11,12]. Third, many animal groups will not have individual uniformity; some groups may have evolved to have specialized leaders and followers [22,24], or these may exist simply because of individual differences in age, experience and dominance hierarchy across the group membership. Similarly, many social groups will have underlying complexity (e.g. familial associations) that may lead to subgroups forming [25,26]. Fourth, with the possible exception of eusocial species, animal groups cannot be assumed to act in the interests of optimizing collective outcomes. Theoretical studies on collective navigation tend to focus on the gains to the group [4], while in unrelated animal groups selection operates on the level of the individual. As such, animals are likely to be adapted to make use of social information to maximize their individual navigational benefits. Work on binary choice decisions [27,28] suggests that individuals may attend more to each other than is optimal for collective estimation. In essence, each individual is motivated to use the available social information, or even information from their own history, once this is more reliable than environmental cues [23,29,30]. While individually rational, this restricts the amount of new information from the environment that can be integrated into the collective estimate.

There are, therefore, several important open questions around the mechanisms of collective navigation. How does collective accuracy depend on the relative weighting individuals place on private information received from the environment, and public social information in the form of the movements of other group members? How should an individually rational agent select a weighting of social information and what are the consequences for collective accuracy? Finally, how might heterogeneous specialized agents emerge within the group, and what is their effect on collective accuracy?

In this article, we describe a general model of collective navigation where agents repeatedly balance environmental and social signals as they navigate. Motivated by examples of long-distance migration [1,2], we assume the target is situated on a distant horizon line, which enables individual estimates to be treated as lying on the real line; combined with a focus on the equilibrium properties of collectively navigating groups rather than the dynamics this facilitates a mathematically tractable analysis. We derive expressions for the long-term navigational accuracy for different weightings of environmental and social information, and identify the collectively optimal weighting of these information sources as a function of group size and information precision. We show how individually rational selection of weightings leads to collectively sub-optimal choices, and how relaxing the assumption of identical agents leads to the emergence of specialization and heterogeneous navigational strategies where agents trade off the costs of information and navigational errors.

## 2. Results

### 2.1. Model outline

We consider a group of n agents, each estimating the location of a target situated on a distant horizon, represented by a value on the real line. Agents make estimates of the position of this target based on two sources of information: private environmental signals and public social information. Although we do not explicitly model an angular bearing, we implicitly assume that each individual orientates towards their estimate of the target. Restricting the problem to a target on the horizon is realistic for collective groups undertaking long-range migration and is consistent with models that assume angular errors are small [24], since within this regime (i.e. where sin⁡(θ)≃θ ) angular statistics can be well approximated by standard linear methods. Short-range navigation such as localized foraging may involve larger angular deviations that violate these assumptions.

Without loss of generality, we assume that the true position of the target on the real line is 0. At any given time t , each agent i makes an estimate of the target, $\kappa_{t}^{i}$ . Time progresses in discrete steps of arbitrary size of 1; we focus on the equilibrium behaviour of the group that is independent of the underlying timescale.

Agents privately observe noisy signals of the target, with each agent observing a different signal at each time step, $z_{t}^{i}∼𝒩(0,1^{2})$ , where the variance of the signal is chosen to be 1 to set an arbitrary scale. As agents estimate their target they also imperfectly observe the current estimates of other agents: each agent i observes an apparent value of the estimate of another agent j at time t , given by $\lambda_{t}^{ij}∼𝒩(\kappa_{t}^{j},\gamma^{2})$ , or equivalently $\nu_{t}^{ij}∼𝒩(0,\gamma^{2})$ and $\lambda_{t}^{ij}=\kappa_{t}^{j}+\nu_{t}^{ij}$ .

Similar to Codling & Bode [23,29], we assume that at each time step agents revise their target estimates via a linear combination of new signals from the target and the observed estimates of other agents, with a target weighting $w_{t}$ representing the relative importance of the environmental signal,


(2.1) $$\kappa_{t+1}^{i}=w_{t}z_{t}^{i}+\frac{1-w_{t}}{n}\sum_{j}\lambda_{t}^{ij}.$$

We note that the agent treats its own current estimate symmetrically to all others. As well as being a common simplifying assumption that can account for an element of persistence in individual movement direction [29], this is a rational use of the available information by an agent that is aware that itself and others share a common navigational goal, and have acquired their estimates by the same process.

A table of model parameters and definitions is given in table 1.

**Table 1.** Definitions of model variables and parameters. Note variables are subscripted by t to indicate time dependence where appropriate in dynamical equations; unsubscripted variables indicate equilibrium values.

parameter	definition
n	group size
z	environmental signal
w	target weighting
$\kappa^{i}$	estimate of agent i
$\lambda^{i\,j}$	estimate of agent j as observed by agent i
$\nu^{i\,j}$	error in observation of agent j’s estimate by agent i
γ	error s.d. in social observations
σ	target error: the s.d. of estimate around the true value

### 2.2. Optimal target weighting for collective accuracy

We first address a classic question of collective navigation [23,29]: what weighting of environmental and social information produces the greatest accuracy for a typical group member? We answer this question by seeking to minimize the *target error*, σ : the standard deviation of the estimate κ . Since the environmental signals z are unbiased, the expected value of κ will always be 0, corresponding to the true location of the target; the standard deviation of κ thus determines the expected root mean square error in the heading. The linear update rule for individual estimates implies a corresponding update rule for target error via the addition rules for correlated variances as given by


(2.2) $$\begin{array}{rl} \text{var}\;(\kappa_{t+1}^{i})=w_{t}^{2}\text{var}(z_{t}^{i}) & +\frac{(1-w_{t}{)}^{2}}{n^{2}}\sum_{j=1}^{n}\sum_{k=1}^{n}\text{cov}(\kappa_{t}^{j},\kappa_{t}^{k})\\ & +\frac{(1-w_{t}{)}^{2}}{n^{2}}\sum_{j=1}^{n}\text{var}(\nu^{ij}). \end{array}$$

We assume that the group is homogeneous such that $\text{var}(\kappa_{t}^{i})=\text{var}(\kappa_{t}^{j})\forall i,j$ . We define $\sigma_{t}^{2}=\text{var}(\kappa_{t}^{i})$ as the variance of an agent’s estimate at time t and $\rho_{t}=\frac{\text{cov}(\kappa_{t}^{i},\kappa_{t}^{j})}{\text{var}(\kappa_{t}^{i})}$ as the correlation between different agents’ estimates. With these definitions, the expression above can be rewritten as


(2.3) $$\sigma_{t+1}^{2}=w_{t}^{2}+\frac{(1-w_{t}{)}^{2}}{n}\;(\sigma_{t}^{2}(1+(n-1)\rho_{t}))+\frac{(1-w_{t}{)}^{2}}{n}\gamma^{2}.$$

The value of $\rho_{t}$ in turn is given by inspection of the shared component of equation (2.2) as:


(2.4) $$\rho_{t}=\frac{\text{cov}(\kappa_{t}^{i},\kappa_{t}^{j})}{\text{var}(\kappa_{t}^{i})}=\frac{\sigma_{t-1}^{2}}{\sigma_{t}^{2}}\;\frac{(1+(n-1)\rho_{t-1})(1-w_{t-1}{)}^{2}}{n}.$$

Since we are interested in long-range animal navigation, we aim to minimize the target error in the long term over many iterations of estimate revision, which we approximate by the equilibrium condition $\sigma_{t+1}=\sigma_{t}$ . Inserting this condition into equations (2.3) and (2.4), we obtain an equilibrium relation between the target error and the target weighting w ,


(2.5) $$\sigma^{2}=\frac{nw^{2}+(1-w{)}^{2}\gamma^{2}}{n-(1-w{)}^{2}\left(1+(n-1)\rho \right)},$$

where ρ is the equilibrium correlation between agents’ estimates, given by


(2.6) $$\rho =\frac{(1-w{)}^{2}}{n-(1-w{)}^{2}(n-1)}.$$

**Figure 1.**
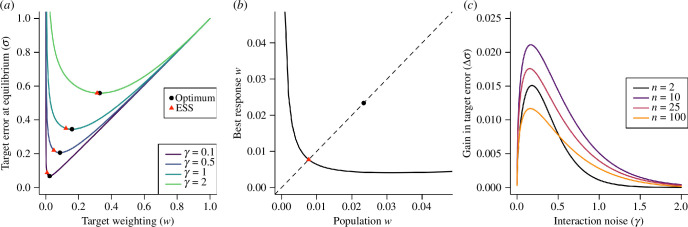
Collectively optimal and evolutionarily stable navigation strategies. (*a*) The equilibrium target error ( σ ) as a function of target weighting ( w ) in a group of 10 agents, for different values of the interaction noise ( γ ). Each curve displays a clear optimal value of w that minimizes the target error, marked by a black circle. The ESS target weighting is shown in each case by a red triangle; this is consistently a lower target weighting than the collectively optimal strategy and leads to a greater target error. (*b*) For any target weighting adopted generally by the group, there is a best-response value of w an individual can employ to minimize its own target error (black line).The ESS occurs where this best response is equal to the population value (red triangle); the collectively optimal target weighting (black circle) is greater than under the ESS and does not lie on the best-response curve. (*c*) The difference between the target error achieved at the ESS and that of the collectively optimal target weighting as a function of the interaction noise for different group sizes, showing a peak for low values of γ .

Given this relationship, we can evaluate σ as a function of w for any given values of γ and n . In figure 1*a* we show this for a range of values of γ with a fixed group size of n=10 . As this plot shows, for each value of γ there is a unique optimal value of w that minimizes σ , which we denote by $w^{*}$ , and thus gives the greatest average accuracy for the individual estimates. In electronic supplementary material, figure S4, we further show how this optimal target weighting varies with the group size n and the interaction noise γ . For a fixed value of γ , the optimal target weighting decreases as the group size increases. For any given value of n , the optimal value of w increases monotonically with γ , and asymptotically approaches one as γ→∞ and zero as γ→0 . The target error obtained by selecting the optimal target weighting is shown in figure 1*c*; as intuitively expected, larger group sizes with less interaction noise are more accurate. The target error σ tends to one as γ→∞ , as individuals attend only to the environmental signal, which is defined to have unit variance. These results are consistent with earlier numerical results [23,29] and are confirmed in simulations of the navigational process over 200 time steps for 2000 random initial conditions, showing the convergence to the same equilibrium condition (electronic supplementary material, figures S1,S2). In electronic supplementary material, appendix A, we formally show that recursive iteration of the model leads to this equilibrium condition as t→∞ for values of w>0 .

### 2.3. Individually rational strategy

In the analysis above, we considered the value of the target weighting that would minimize the target error, assuming that this value would be used by all agents. However, in groups of unrelated individuals, selective pressure and individual rationality will push individuals to adopt strategies that maximize their individual accuracy, not necessarily that of other agents as well [31]. It is important, therefore, to consider whether the optimal target weighting derived above represents not only a collective optimum but also an evolutionary stable state (ESS) of the system [28].

Consider a group in which all agents currently employ the same target weighting, w . We can now consider what target weighting $w^{\prime }$ an individual agent should adopt to minimize its target error, assuming that all other agents continue to use w . From equation (2.3), we have


(2.7) $$\sigma^{\prime 2}=w^{\prime 2}+\frac{(1-w^{\prime }{)}^{2}}{n}\;(\sigma^{2}(1+(n-1)\rho ))+\frac{(1-w^{\prime }{)}^{2}}{n}\gamma^{2},$$

where $\sigma^{\prime }$ is the target error for the focal agent, and σ and ρ are the target error and correlation of the other agents’ estimates resulting from the use of w . The rational choice for the focal agent is that which minimizes $\sigma^{\prime }$ , thus satisfying


(2.8) $$\frac{\partial \sigma^{\prime 2}}{\partial w^{\prime }}=0.$$

This implies a solution for $w^{\prime }$ :


(2.9) $$w^{\prime }=\frac{\sigma^{2}(1+(n-1)\rho ))+\gamma^{2}}{n+\sigma^{2}(1+(n-1)\rho ))+\gamma^{2}}.$$

Figure 1*b* shows an example of ‘best-response’ values of $w^{\prime }$ by a focal agent minimizing its own target error, responding to a population employing a target weighting w . There is a fixed point where the best response equals the current target weighting employed by the population, which represents an ESS; when all agents adopt this target weighting, any individual deviating from this value will increase its own target error. Figure 1*a* shows the relationship between the population target weighting and target error for a range of interaction noise values, along with both the optimal target weighting and the ESS target weighting; the ESS value of the target weighting is always lower than the collectively optimal value and leads to greater target error (see electronic supplementary material, appendix B, figure S3 for further mathematical analysis of this relationship).

As shown in figure 1*c*, the absolute increase in target error due to adoption of the ESS target weighting (relative to the optimal target weighting) is greatest at low values of γ , though it declines for very low values of γ as the optimal target weighting also approaches zero. The relationship with group size is complicated; the increase in σ from adopting the ESS is not monotonic, but first increases with group size before declining for larger groups, due to the optimal target weighting approaching zero for large n (see electronic supplementary material, figure S4).

### 2.4. Mixed-expertise groups

So far we have considered groups of identical agents who receive environmental signals of equal precision and who are indistinguishable (i.e. a focal agent attends to all other agents equally). However, much research on collective motion has focused on the role of leadership and hierarchies in navigation, including their connection to individuals’ differing expertise and private knowledge of the target [7,8,17,32,33]. We now consider a group composed of two types of individuals, type A ‘expert’ agents and type B ‘naive’ agents that differ in the precision of the environmental signal they receive: type A agents receive signals z∼N(0,1) , while type B agents receive signals with a greater variance $z∼N(0,\epsilon^{2})$ ( ϵ>1 ). Furthermore, we assume that agents can distinguish individuals of the two types (for instance, by the differing size and markings of juvenile individuals relative to mature adults). Since here we are concerned only with differing expertise regarding the environmental signal, we assume that the precision with which agents can observe each other ( γ ) is uniform across all individuals. Importantly, although expertise often correlates with leadership [17], here we do not assume *a priori* that either type of agent should adopt a particular social role in the group, such as a ‘leader’ or ‘follower’, only that these two types of agents exist in the population, and each individually seeks to minimize its target error.

Each agent is thus faced with a decision on how to weight three potential sources of information: (i) their own private information z ; (ii) the current headings of type A individuals; and (iii) the current headings of type B individuals. We parametrize this choice via the weight given to private information ( w ) and the relative weight given to following experienced individuals over naive individuals ( α ). Since agents of different types can be expected to weight each information source differently, we allow for different parameters for agents of each type: $w_{A},w_{B}$ and $\alpha_{A},\alpha_{B}$ . Since we will seek to derive the optimal values of these parameters at equilibrium we take these to be fixed values through time. The update rule for each type A agent’s new heading is thus


(2.10) $$\kappa_{t+1}^{i}=w_{A}z_{t}^{i}+(1-w_{A})\;\left[\frac{\alpha_{A}}{n_{A}}\sum_{j\in A}\lambda_{t}^{ij}+\frac{\left(1-\alpha_{A}\right)}{n_{B}}\sum_{k\in B}\lambda_{t}^{ik}\right],$$

where $z∼N(0,1^{2})$ , $\lambda_{t}^{j}∼N(\kappa_{t}^{j},\gamma^{2})$ and $\alpha_{A}$ is a weighting for following other type A agents rather than type B agents. Similarly, type B agents update as


(2.11) $$\kappa_{t+1}^{i}=w_{B}z_{t}^{i}+(1-w_{B})\;\left[\frac{\alpha_{B}}{n_{A}}\sum_{j\in A}\lambda_{t}^{ij}+\frac{\left(1-\alpha_{B}\right)}{n_{B}}\sum_{k\in B}\lambda_{t}^{ik}\right],$$

with $z∼N(0,\epsilon^{2})$ . Given the update rule above, the corresponding equilibrium equations for the target errors of each type of agent are given by


(2.12) $$\begin{array}{rl} \sigma_{A}^{2} & =w_{A}^{2}\\ & +(1-w_{A}{)}^{2}((\alpha_{A}^{2}/n_{A})\sigma_{A}^{2}(1+(n_{A}-1)\rho_{AA}))\\ & +(1-w_{A}{)}^{2}(((1-\alpha_{A}{)}^{2}/n_{B})\sigma_{B}^{2}(1+(n_{B}-1)\rho_{BB}))\\ & +2(1-w_{A}{)}^{2}((\alpha_{A}(1-\alpha_{A}))\sigma_{A}\sigma_{B}\rho_{AB})\\ & +(1-w_{A}{)}^{2}\;\left(\frac{\alpha_{A}^{2}}{n_{A}}+\frac{(1-\alpha_{A}{)}^{2}}{n_{B}}\right)\;\gamma^{2}\\ \sigma_{B}^{2} & =\epsilon^{2}w_{B}^{2}\\ & +(1-w_{B}{)}^{2}((\alpha_{B}^{2}/n_{A})\sigma_{A}^{2}(1+(n_{A}-1)\rho_{AA}))\\ & +(1-w_{B}{)}^{2}(((1-\alpha_{B}{)}^{2}/n_{B})\sigma_{B}^{2}(1+(n_{B}-1)\rho_{BB}))\\ & +2(1-w_{B}{)}^{2}((\alpha_{B}(1-\alpha_{B}))\sigma_{A}\sigma_{B}\rho_{AB})\\ & +(1-w_{B}{)}^{2}\;\left(\frac{\alpha_{B}^{2}}{n_{A}}+\frac{(1-\alpha_{B}{)}^{2}}{n_{B}}\right)\;\gamma^{2}, \end{array}$$

where $\rho_{AA}$ is the correlation between the headings of type A agents, and similarly $\rho_{BB}$ is the correlation between the headings of type B agents and $\rho_{AB}$ is the cross-correlation between headings of type A and type B agents. These values are given by the following equilibrium equations:


(2.13) $$\begin{array}{rl} \rho_{AA}= & \frac{(1-w_{A}{)}^{2}}{\sigma_{A}^{2}}\times \\ & [\left(\alpha_{A}^{2}/n_{A})\sigma_{A}^{2}(1+\rho_{AA}(n_{A}-1)\right)\\ & +((1-\alpha_{A}{)}^{2}/n_{B})\sigma_{B}^{2}(1+\rho_{BB}(n_{B}-1))\\ & +2\alpha_{A}(1-\alpha_{A})\sigma_{A}\sigma_{B}\rho_{AB}],\\ \rho_{BB}= & \frac{(1-w_{B}{)}^{2}}{\sigma_{B}^{2}}\times \\ & [\left(\alpha_{B}^{2}/n_{A})\sigma_{A}^{2}(1+\rho_{AA}(n_{A}-1)\right)\\ & +((1-\alpha_{B}{)}^{2}/n_{B})\sigma_{B}^{2}(1+\rho_{BB}(n_{B}-1))\\ & +2\alpha_{B}(1-\alpha_{B})\sigma_{A}\sigma_{B}\rho_{AB}]\\ \;\text{and}\;\rho_{AB}= & \frac{\left(1-w_{A})(1-w_{B}\right)}{\sigma_{A}\sigma_{B}}\times \\ & [\left(\alpha_{A}\alpha_{B}/n_{A})\sigma_{A}^{2}(1+\rho_{AA}(n_{A}-1)\right)\\ & +((1-\alpha_{A})(1-\alpha_{B})/n_{B})\sigma_{B}^{2}(1+\rho_{BB}(n_{B}-1))\\ & +(\alpha_{A}(1-\alpha_{B})+\alpha_{B}(1-\alpha_{A}))\sigma_{A}\sigma_{B}\rho_{AB}]. \end{array}$$

To solve for the equilibrium target errors $\sigma_{A}$ and $\sigma_{B}$ for fixed parameter values $w_{A},w_{B},\alpha_{A},\alpha_{B}$ , we treat equations (2.12) and (2.13) as update equations, initializing with a state of $\rho_{AA}=\rho_{BB}=\rho_{AB}=0$ , $\sigma_{A}=1$ and $\sigma_{B}=\epsilon$ (representing an initial orientation based on purely environmental signals), and iterating until convergence.

To demonstrate the predictions of this model, we consider a simple case where a group of 10 individuals has one experienced agent with better environmental information than the others, and we explore both the collectively optimal and ESS values of target weighting and social weighting in individuals of the two types as a function of the variance of the follower’s environmental signal. Since agents of different types will not necessarily experience the same target error, to define collective optimality we must choose some function of all agents’ target errors that represents the collective performance to be optimized. We choose this to be the root mean square target error of all agents, such that


(2.14) $$\text{collective target error}=\sqrt{\frac{n_{A}\sigma_{A}^{2}+n_{B}\sigma_{B}^{2}}{n_{A}+n_{B}}}.$$

The collectively optimal strategy consists of the values $w_{A},w_{B},\alpha_{A}$ and $\alpha_{B}$ that minimize this collective target error measure, which we identify by standard Nelder–Mead numerical optimization [34].

The ESS strategy can be derived similarly to the case of the homogeneous group, by considering the best response values of $w_{A}^{\prime },\alpha_{A}^{\prime }$ ( $w_{B}^{\prime },\alpha_{B}^{\prime }$ ) that minimize the target error of a type A (type B) agent, conditioned on the current values employed by the rest of the group. The ESS strategy is then defined by values of $w_{A},w_{B},\alpha_{A},\alpha_{B}$ such that no agent can reduce its target error by deviating from these values. This implies that the following identities hold:


(2.15) $$\frac{\partial \sigma_{A}^{\prime 2}}{d\alpha_{A}^{\prime }}=\frac{\partial \sigma_{B}^{\prime 2}}{d\alpha_{B}^{\prime }}=\frac{\partial \sigma_{A}^{\prime 2}}{dw_{A}^{\prime }}=\frac{\partial \sigma_{B}^{\prime 2}}{dw_{B}^{\prime }}=0.$$

Taking the appropriate derivatives to identify these minima gives


(2.16) $$\alpha_{A}^{\prime }=\alpha_{B}^{\prime }=\frac{\zeta_{B}}{\zeta_{A}+\zeta_{B}},$$

where


(2.17) $$\begin{array}{rl} \zeta_{A} & =\sigma_{A}^{2}(1+(n_{A}-1)\rho_{AA}))-\sigma_{A}\sigma_{B}\rho_{AB}-\gamma^{2}/n_{A}\\ \zeta_{B} & =\sigma_{B}^{2}(1+(n_{B}-1)\rho_{BB}))-\sigma_{A}\sigma_{B}\rho_{AB}-\gamma^{2}/n_{B}. \end{array}$$

Similarly, the updated target weightings are given by


(2.18) $$w_{A}^{\prime }=\frac{\psi_{A}}{1+\psi_{A}},\;w_{B}^{\prime }=\frac{\psi_{B}}{\epsilon +\psi_{B}},$$

where:


(2.19) $$\begin{array}{rl} \psi_{A}= & \left(\alpha_{A}^{\prime 2}/n_{A})\sigma_{A}^{2}(1+(n_{A}-1)\rho_{AA}\right)\\ & +(1-\alpha_{A}^{\prime }{)}^{2}/n_{B})\sigma_{B}^{2}(1+(n_{B}-1)\rho_{BB})\\ & +2((\alpha_{A}^{\prime }(1-\alpha_{A}^{\prime }))\sigma_{A}\sigma_{B}\rho_{AB})\\ & +\gamma^{2}/(n_{A}+n_{B})\\ \psi_{B}= & \left(\alpha_{B}^{\prime 2}/n_{A})\sigma_{A}^{2}(1+(n_{A}-1)\rho_{AA}\right)\\ & +(1-\alpha_{B}^{\prime }{)}^{2}/n_{B})\sigma_{B}^{2}(1+(n_{B}-1)\rho_{BB})\\ & +2((\alpha_{B}^{\prime }(1-\alpha_{B}^{\prime }))\sigma_{A}\sigma_{B}\rho_{AB})\\ & +\gamma^{2}/(n_{A}+n_{B}). \end{array}$$

These best-response equations specify updates to the parameter values that we iterate until we reach equilibrium to find the stable values of $w_{A},w_{B}$ and $\alpha_{A},\alpha_{B}$ as well as the resulting target errors for agents of each type. We initialize with values of $w_{A}=w_{B}=1$ , $\alpha_{A}=\alpha_{B}=1/2$ and $\rho_{AA}=\rho_{BB}=\rho_{AB}=0$ , representing an initial state in which agents exclusively attend to their private environmental cue and, therefore, have no initial correlation in their headings.

**Figure 2.**
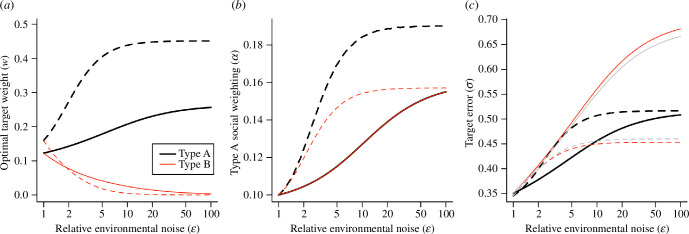
Collectively optimal (dashed lines) and evolutionary stable state (solid lines) in groups composed of one experienced individual and nine naive individuals, as a function of the relative noise level ( ϵ ). Naive individuals receive target signals with variance $\epsilon^{2}$ , the experienced individual receives target signals with variance 1. (*a*) As ϵ increases, naive individuals reduce their target weighting, with $w_{B}$ tending to zero for very high values of ϵ , while ‘expert’ agents increase their target weighting. In the collectively optimal strategy, type A agents direct substantially more attention to the target signal. (*b*) Both expert and naive agents attend more to the expert agent as ϵ increases. In the ESS, agents of both types adopt identical values of α , the relative weighting for following the experienced agent over the naive agents. (*c*) The target error increases with increasing values of ϵ . In the ESS, the superior private information of the experienced individual is reflected in a lower target error ( σ ); this difference increases with ϵ . However, in the collectively optimal strategy, the type A agent has a higher target error. The target error for each strategy is shown in grey and is substantially greater under the ESS than under the collectively optimal strategy for high values of ϵ .

Figure 2 shows the values of the collectively optimal (dashed lines) and ESS (solid lines) parameters for type A and type B agents as a function of the relative environment noise level ϵ . As ϵ increases the naive agents attend less and less to their environmental signal, with $w_{B}$ approaching zero (figure 2*a*), while the expert agent increases its target weighting to compensate for the increasingly unreliable social information from other agents. This effect is stronger in the collectively optimal strategy than the ESS, showing that it is useful for the group to have the expert agent attend strongly to environmental signals for the benefit of all. We might intuitively expect that in this case, the members of the group would transition to exclusively following the one experienced agent with a good environmental signal. However, while $\alpha_{A}$ and $\alpha_{B}$ increase with ϵ under both the collectively optimal and ESS strategies, they remain substantially below 1, indicating that agents of both types continue to attend to the headings of type B agents even for very high values of ϵ (figure 2*b*). This shows that even in cases where naive agents have no useful environmental signal, both the experienced agent and the naive agents embed useful navigational information in their current estimates (cf. [23,29]). In the ESS, both types of agent attend equally to the heading of the type A individual (relative to other agents), and both do so less than would be collectively optimal. Under the ESS, the target error of the naive agents is higher than that of the experienced agent (figure 2*c*); this difference becomes substantial as ϵ becomes large, reflecting the low accuracy of the naive agents’ private information. The grey lines in figure 2*c* indicate the collective target error, which is by definition the same for agents of both types; as ϵ becomes large the collective target error is lower than that of either type of agent under the ESS, showing that groups composed of agents with many uninformed individuals pay a substantial penalty in terms of navigational accuracy if agents seek only to optimize their own individual accuracy.

### 2.5. Emergence of leader and follower strategies

In the above analysis of mixed-experience groups, we assumed that differences in the environmental signal were due to fixed inherent differences in the quality of private information individuals of two types received. Our results showed that under such circumstances agents with very poor private information were substantially less accurate than those with accurate private information. This suggests that even if private information is costly, agents may gain an advantage by paying this cost to improve their navigational accuracy. However, there is also the opportunity to free-ride by following others if private information imposes costs in attention or maintenance of sensory apparatus, and if other agents are already attending to good environmental signals. This can present another potential source of heterogeneity in groups: the adoption of differing navigational ‘strategies’, such as ‘leaders’ and ‘followers’. The emergence of such strategies has been demonstrated theoretically in the context of other navigational models [22,24]. A natural question is whether our model also supports the evolution of heterogeneous strategies.

To explore this, we consider groups in which each agent has an individually specific target weighting ( w ), variance of environmental noise ( $\epsilon^{2}$ ) and variance of social information ( $\gamma^{2}$ ). Following the general approach of Guttal & Couzin [22], we impose fitness costs on the precision of both environmental and social information, such that the fitness of agent i , $F_{i}$ , is given by


(2.20) $$F_{i}=-\sigma_{i}-1/\epsilon_{i}^{k}-1/\gamma_{i}^{k},$$

where k is an adjustable parameter that allows for differing information cost profiles. This is of course only one possible fitness function, but it is one that will allow us to explore scenarios where heterogeneity does or does not emerge. We note the importance of assigning costs to precision in both environmental and social information. If environmental information can be obtained with precision at no cost then agents can navigate perfectly by attending solely to the target. Conversely, if the precision of social information carries no cost then the variance parameter γ can equal zero; as shown earlier, this limiting case results in an unrealistic situation in which the best choice of target weighting tends to zero, and the convergence of the group to a stable target error becomes very slow.

If each agent instantly adopts an optimal value for its parameters (maximizing fitness) at any given time then symmetry demands that all agents will converge on the same parameters. However, in reality, it is likely that agents will adapt their parameters locally and gradually, whether by evolutionary adaptation or learning. To simulate this, we allow each agent at each time step to trial a random local mutation of its current parameter values, which we initialize with random starting values. If the trial values produce a greater fitness the agent adopts them, otherwise, it retains its current values.

As in the case of a homogeneous group, each agent updates its heading by a linear combination of a target signal $z_{i}$ (with variance $\epsilon_{i}^{2}$ ) and the perceived average heading of all group members. The agent also has its own target weighting $w^{i}$ , which specifies how heavily it weights the target signal in this update step. Thus, for agent i we have


(2.21) $$\kappa_{t+1}^{i}=w_{t}^{i}z_{t}^{i}+\frac{1-w_{t}^{i}}{n}\sum_{j}\kappa_{t}^{j}+\frac{1-w_{t}^{i}}{n}\sum_{j}\nu_{t}^{ij},$$

where $z_{t}^{i}∼N(0,\epsilon_{i}^{2})$ and $\nu_{t}^{ij}∼N(0,\gamma_{i}^{2})$ . The variance of the average heading is given by summing over the covariance matrix for all current headings


(2.22) $$\text{var}\;\left(\frac{1}{n}\sum_{j}\kappa_{t}^{j}\right)=\frac{1}{n^{2}}\;\left(\sum_{k}\sum_{l}\text{cov}(\kappa_{t}^{k},\kappa_{t}^{l})\right).$$

The elements of the covariance matrix are in turn given by


(2.23) $$\text{cov}(\kappa_{t+1}^{k},\kappa_{t+1}^{l})=(1-w_{k})(1-w_{l})\text{ var}\;\left(\frac{1}{n}\sum_{j}\kappa_{t}^{j}\right)+\gamma_{k}^{2}\delta_{k,l},$$

where $\delta_{k,l}$ is the Kronecker delta.

Combining these elements provides the update equation for the target error of agent i


(2.24) $$\sigma_{t+1}^{2}=w_{i}\epsilon_{i}^{2}+\frac{(1-w_{i}{)}^{2}}{n^{2}}\;\left(\sum_{k}\sum_{l}\text{cov}(\kappa_{t}^{k},\kappa_{t}^{l})\right)+\frac{(1-w_{i}{)}^{2}}{n}\gamma_{i}^{2}.$$

We initialize all agents with random initial values from


(2.25) w∼U(0,1), ϵ∼exp(1), γ∼exp(1).

At each subsequent time step, we choose a random agent to test a mutation of these parameters, generated as


(2.26) $$\begin{array}{rl} & w^{\prime }∼N(w,0.01^{2})\;\text{[bounded between 0 and 1]},\\ & \log\epsilon^{\prime }∼N(\log\epsilon ,0.01^{2})\;\text{and}\\ & \log\gamma^{\prime }∼N(\log\gamma ,0.01^{2}) \end{array}$$

We evaluate the new target error for this agent under the mutated parameters (assuming that all other agents’ parameters remain fixed), and if this is lower than under the previous parameters the mutation is accepted, otherwise it is rejected. We repeat this process for n×50000 time steps.

**Figure 3.**
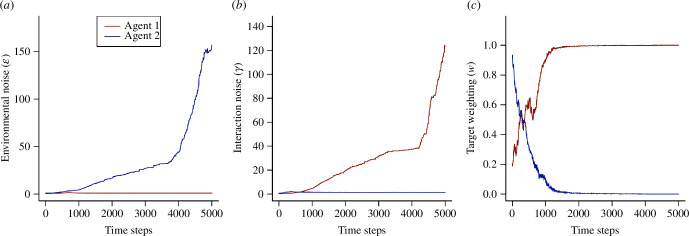
Example of the evolution of heterogeneous strategies in a paired ( n=2 ) navigation scenario. Each curve shows the evolution of one parameter of the model in one of two agents, with the information cost parameter being fixed at k=1 . (*a*) The evolution of the environmental noise parameter ( ϵ ). (*b*) The evolution of the interaction noise parameter ( γ ). (*c*) The evolution of the target weighting ( w ). One agent evolves a low value of ϵ , a high value of γ and a high value of w , indicating a ‘leading’ strategy, focused on attending to environmental cues; the other agent adopts a ‘following’ strategy with high ϵ , low γ and low w , focused on accurately observing and following the leader.

**Figure 4.**
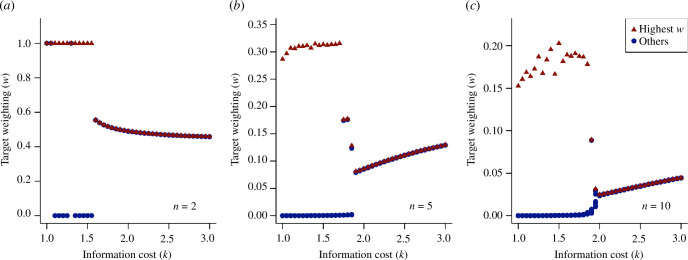
ESS target weighting for agents navigating in a group with costly information, as a function of the information cost parameter k , for group sizes of (*a*) 2; (*b*) 5 and (*c*) 10. Below a critical value of k , the agents typically diverge into distinct strategies with one agent adopting the role of ‘leader’ (highest value of w , red triangles) and the others ‘followers’ (lower values of w , blue circles). For higher values of k , the agents converge on a single common strategy with an intermediate target weighting. Points represent the final target weighting for all agents after 50000×n time steps, with agents randomly initialized for each value of k .

Using a value of k=1 in groups of n=2 agents, we see a tendency for the system to evolve to a heterogeneous state, where one individual becomes the ‘leader’ and the other the ‘follower’, visible in the evolution of the individual parameter values (figure 3). The leader is characterized by a high precision of target information (low ϵ ), a low precision of social information (high γ ) and a high target weighting ( w≃1 ), indicating that it relies on good quality environmental information and ignores the other agent. Conversely, the follower has low precision of target information (high ϵ ), high precision of social information (low γ ) and a low target weighting ( w≃0 ), indicating a strategy of keenly observing and following the other agent. Electronic supplementary material, figure S5 shows the outcome of 10 further simulations, demonstrating that the emergence of a leader and follower is typical, but not inevitable. In some cases, both agents evolve high interaction noise at an early stage; this effectively makes each an independent navigator and thus both retain high target weightings. Across 1000 randomly initiated trials, in 734 cases the agents split into these leader–follower strategies. In the remaining 266 cases, both agents evolved high target weightings ( w≃1 ). Different values of k alter the trade-off between the cost of information and the cost of navigational inaccuracy, and therefore can lead to different strategies emerging. Performing the same analysis over a range of values for k reveals a critical value below which the agents diverge into leader and follower roles, and above which they evolve towards a common strategy, with the critical value being k≃1.5 . Figure 4*a* shows these results for one simulation at each value of k . We stress that our choice of the fitness function is arbitrary, so no special significance should be given to the precise value of this bifurcation point, but its existence illustrates how the costs of different sources of information can determine whether groups navigate democratically or through a subset of leaders. These results are not specific to groups of two agents; the same analysis on larger groups reveals that this bifurcation is a general phenomenon across group sizes (figure 4*b*,*c*). In all cases, where k is sufficiently small, there exists an ESS in which one agent adopts the role of ‘leader’ and maintains an appreciable attention ( w ) to the environmental signal ( z ), while all other agents become ‘followers’ ( w→0 ). In groups of n=2 , an alternative equilibrium is sometimes reached, in which both agents retain high target weighting; in groups of n=5 and n=10 a single leader always emerges. Electronic supplementary material, figure S6 shows the distribution of target weights over 100 simulations, illustrating the repeatability of this result. As expected from our earlier results, in larger groups the value of w for the leader is lower. The regular emergence of a single leader in this model likely reflects the assumption that each agent can observe the current estimates of all others in the group, implying that a single leader can be followed by all other agents. Other models that assume spatially localized neighbour-following rules typically give rise to more than one leader, such that these leaders can be observed and followed by all other members of the group.

## 3. Discussion

Our findings indicate that the optimal long-term accuracy for group navigation is achieved when agents pay minimal attention to immediate environmental cues, and instead strongly follow their current direction and those of group members [23,29]. These current directions essentially form a collective memory of past environmental signals, helping agents to avoid overreacting to new, potentially noisy signals. However, when perceiving current directions becomes challenging, greater environmental attention becomes necessary.

Our model demonstrates the qualitative features of the ‘many wrongs’ hypothesis: larger groups are more accurate as they pool more information [19,20]. However, the accuracy gains are limited by several factors. Noisy observations of other agents limit how well information can be aggregated, and when agents can accurately observe each other the correlations that emerge between agents reduce the effective number of independent samples that the group represents. Collective accuracy is also limited by the instability of collectively optimal navigation strategies to invasion by individuals who utilize environmental information less and social information more. This has the effect of reducing the amount of new environmental information entering the group and thus reduces the amount of information about the target that can be pooled.

We also studied the difference between a collectively optimal strategy and an individually rational one (an ESS), a central concept in economic and evolutionary theory and one that has been explored in the context of collective decision-making [27,28], with results showing that individual selection tends to favour stronger social responses than are collectively optimal. We found that groups composed of self-interested individuals who only seek to optimize their individual accuracy will navigate less effectively as a result of each agent attempting to ‘free-ride’ on the navigational efforts of others, such that the group collectively attends less to environmental cues than would otherwise be optimal for accurate navigation. The magnitude of this deviation from optimality is predicted to be largest in the regime where interaction noise is low, i.e. where agents can accurately perceive each other, and where as a consequence the optimal target weighting is already low. However, although such conditions maximize the absolute difference between optimal and evolutionary stable navigation strategies, within this regime both strategies are highly accurate, with the average target error becoming negligible as the interaction noise becomes very small. Therefore, we can predict that individual selection effects will not prevent such groups from navigating accurately.

Much recent work on collective motion has focused on leadership within groups and its origins in individual heterogeneity among group members [16,17,35]. Leadership in the form of unequal influence on collective motion has been identified empirically across a variety of very different taxa, including species of fish [36,37], birds [7,8,32] and mammals [38]. The underlying reasons why some individuals have and exercise this influence have been debated, with factors such as dominance hierarchies [38], personality [36,37,39,40] and individual navigation accuracy [8,33] all being identified as correlates of leadership. We have shown that where differences in perceptual ability are exogenous to the navigational process itself (for instance, resulting from differential maturity and experience), this has a substantial impact on how much agents should attend to environmental rather than social cues, and on which agents they should preferentially follow. As intuitively expected, agents with superior expertise provide more useful social information, but nonetheless even naive agents provide an important navigational function, acting as a ‘buffer’ that stores previously received environmental cues, creating a ‘historical many wrongs’ effect in which the group is able to integrate many past estimates without any agent maintaining an explicit individual memory [29].

When both environmental and social cues are costly to obtain with high precision, leadership can emerge endogenously as the result of individuals seeking to minimize their target error while also minimizing the cost of information. Under these conditions, and for specific information cost functions, agents tend to naturally evolve into distinct leader and follower phenotypes, such that some pay high costs for accurate environmental information, which they then follow and ignore social cues, while others conversely pay high costs for accurate social cues, follow the group and ignore environmental signals. This result mirrors previous demonstrations of the emergence of leader/follower phenotypes in models based on self-propelled particles [22] and stochastic differential equations [24]. A key difference in our approach is that we treat both signals from the environment and social cues explicitly as sources of information, rather than ‘forces’ that act on the agents, which more accurately reflects the cognitive reality of animal navigators. This allows us to differentiate between the precision of sensory cues and the weighting the individual places on each cue. Making this distinction means we do not impose a negative correlation between the precision of environmental and social sensing—instead, this emerges naturally from the evolutionary dynamics. Whether or not groups exhibit this diversity of strategies in reality will depend on the cost of information of different types and the inherent behavioural plasticity of the species in question.

The model we have developed and analysed is motivated by mathematical tractability. As such we have made a number of assumptions that introduce limitations to the interpretation of our findings. First, our results are based on a linear statistical analysis that ignores the effects of angular statistics. While we expect that the effects we have explored will remain qualitatively consistent across spatial scales, our mathematical derivations are strictly limited to cases where this linearization is accurate, such as long-range migrations towards distant targets. Where migrations can be decomposed into multiple stages between waypoints, our conclusions remain applicable within each stage that is itself long range. However, deviations from our expectations are likely at the last stages of such migrations, or in very short-range tasks such as local foraging. Second, we have assumed that all group members can observe and respond to each other. This is in contrast to models in which agents observe only those ‘neighbours’ within a local area or topological distance. As noted earlier, this is likely to introduce variations in the number of leaders that are likely to evolve and that are necessary to accurately lead a group. However, if the network of social connections in a group contains no isolated subgroups we expect our conclusions to remain qualitatively similar in these cases. Finally, in our model, agents do not distinguish between their own current estimate and those of other agents when responding to social information. This presupposes that agents are not despotic and unwilling to compromise with others. This is a natural consequence of assuming that agents are rational and share a common navigational target. Further studies may consider groups of agents with different navigational goals or preferences [41], and these would need to relax this assumption.

## Data Availability

R code to reproduce the analyses in this paper is available as electronic supplementary material [42].
